# Phosphorylation-Mediated Control of Histone Chaperone ASF1 Levels by Tousled-Like Kinases

**DOI:** 10.1371/journal.pone.0008328

**Published:** 2009-12-16

**Authors:** Maxim Pilyugin, Jeroen Demmers, C. Peter Verrijzer, Francois Karch, Yuri M. Moshkin

**Affiliations:** 1 Department of Zoology and National Research Center Frontiers in Genetics, University of Geneva, Geneva, Switzerland; 2 Proteomics Center, Erasmus University Medical Center, Rotterdam, The Netherlands; 3 Department of Biochemistry, Center for Biomedical Genetics, Erasmus University, Rotterdam, The Netherlands; Brunel University, United Kingdom

## Abstract

Histone chaperones are at the hub of a diverse interaction networks integrating a plethora of chromatin modifying activities. Histone H3/H4 chaperone ASF1 is a target for cell-cycle regulated Tousled-like kinases (TLKs) and both proteins cooperate during chromatin replication. However, the precise role of post-translational modification of ASF1 remained unclear. Here, we identify the TLK phosphorylation sites for both Drosophila and human ASF1 proteins. Loss of TLK-mediated phosphorylation triggers hASF1a and dASF1 degradation by proteasome-dependent and independent mechanisms respectively. Consistent with this notion, introduction of phosphorylation-mimicking mutants inhibits hASF1a and dASF1 degradation. Human hASF1b is also targeted for proteasome-dependent degradation, but its stability is not affected by phosphorylation indicating that other mechanisms are likely to be involved in control of hASF1b levels. Together, these results suggest that ASF1 cellular levels are tightly controlled by distinct pathways and provide a molecular mechanism for post-translational regulation of dASF1 and hASF1a by TLK kinases.

## Introduction

Transmission, maintenance and expression of the eukaryotic genomes are mediated by a repertoire of chromatin transactions. A wide range of modifying activities acts on nucleosome, a central unit of chromatin, including histone chaperones, ATP-dependent chromatin remodeling factors (remodelers), and histone modifying enzymes. The accumulated evidence on chromatin modifications reveals extensive role for histone chaperones going beyond just facilitating chromatin assembly [1], [2], [3]. Histone chaperones form extensive interaction networks and in conjunction with other factors participate in a variety of chromatin transactions and cellular tasks [1], [2], [3], [4].

Recent studies have emphasized a critical role for histone H3/H4 chaperone ASF1 in orchestrating distinct chromatin modifying activities. ASF1 hands off histones to CAF1 and HIRA facilitating replication-coupled and replication-independent chromatin assembly respectively [5], [6], [7], [8], [9]. Replication-coupled chromatin assembly also involves a complex of ASF1 with MCM2–7 DNA helicase, which is required for DNA unwinding [7]. In conjunction with Rtt109 and CBP/p300 acetyltransferases, ASF1 stimulates chromatin assembly and incorporation of H3K56Ac marks at DNA repair foci [10], [11]. In addition to chromatin assembly, ASF1 cooperates with SWI/SNF family of ATP-dependent chromatin remodelers and H2A/H2B chaperone FACT to displace nucleosomes from active promoters [12], [13], [14], and in Drosophila ASF1 cooperates *in vivo* with the BRM chromatin remodeling pathway [15]. To further add to the diversity of ASF1 functions, ASF1 is involved in developmental gene expression control [4], [16]. In Drosophila, ASF1 forms a complex with LID/KDM5 H3K4me2/3 demethylase – LAF-A [4]. LAF-A and related LID complexes are tethered to NOTCH target genes by the sequence-specific DNA binding protein Su(H) to mediate gene selective silencing [4], [16].

Given a broad spectrum of ASF1 functions in chromatin regulation, control of ASF1 levels is critical for cell viability, replication and execution of developmental programs. Depletion or overexpression of ASF1 results in severe developmental defects, altered cell cycle progression and cell death [5], [7], [10], [15], [17]. Here, we investigate molecular mechanisms of post-translational regulation of ASF1 levels by a family of conserved serine/threonine Tousled-like kinases (TLKs). ASF1 is a target for cell cycle regulated TLK kinases and both proteins cooperate in control of chromatin replication and cell cycle progression [17], [18], [19]. We identify TLK phosphorylation sites for Drosophila and human ASF1 proteins, and show that loss of TLK activity or mutation of these sites results in degradation of ASF1 by both proteasome-dependent and independent pathways.

## Results

### Mapping of ASF1 Phosphorylation Sites

**Figure 1:**
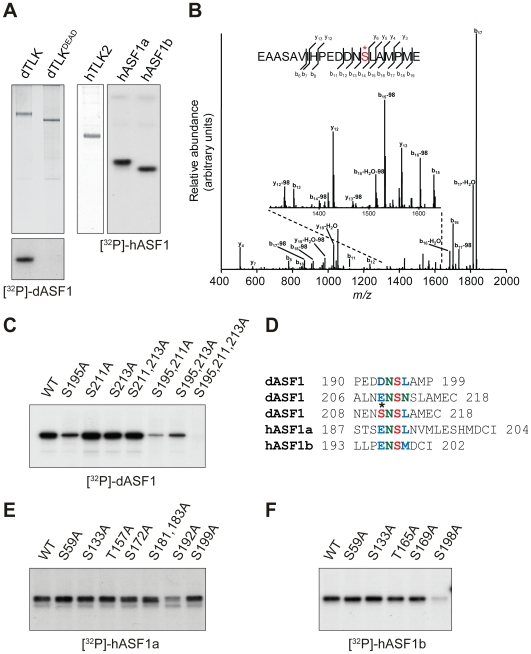
Mapping of ASF1 phosphorylation sites. (**A**) Drosophila TLK, TLKdead and human hTLK2 kinases were expressed in Sf9 cells and purified using His-tag. Phosphorylation of GST-ASF1 proteins by TLKs was performed in the presence of [γ-^32^P]ATP and revealed by autoradiography. dASF1 is phosphorylated by TLK, but not TLKdead (lower panel), and hASF1a and hASF1b can be phosphorylated by hTLK2 (right panel). (**B**) CID fragmentation spectrum of a doubly charged proteolytic peptide of dASF1 (m/z = 1103.96; Mascot identification peptide score  = 88). The peptide carries a phosphorylated serine residue, which is marked with an asterisk. (**C**) *In vitro* phosphorylation of dASF1 mutants by dTLK. S195A alone strongly reduces phosphorylation of dASF1, and phosphorylation of triple mutant S195/211/213A is completely abolished. (**D**) Alignment of ASF1 phosphorylation sites. Asterisk indicates phosphorylated S211 of dASF1 as it structurally resembles Asp (D) or Glu (E) creating optimal target site at S213 for TLK phosphorylation. (**E, F**) *In vitro* phosphorylation of hASF1a and hASF1b mutants by hTLK2. hASF1a S192A (E) and hASF1b S198A (F) mutations strongly reduce phosphorylation efficiency by hTLK2.

In order to understand the importance of TLK-mediated phosphorylation of ASF1, we decided to map the TLK phosphorylation sites in the Drosophila ASF1 protein. For this, bacterially produced dASF1 was phosphorylated in vitro, using dTLK expressed and purified from baculovirus-infected cells (Figure 1A). Mass spectrometry analysis revealed Serine 195 (S195) as a major phosphorylation site within dASF1 (Figure 1B). Mutation of this Serine to Alanine (S195A) significantly reduces dTLK-mediated phosphorylation (Figure 1C). Because phosphorylation was not completely inhibited, we mutated two additional Serines with similar neighboring sequences (S211 and S213) that were not covered by our mass-spectrometry analysis (Figure 1D). Although, all mutated dASF1 proteins retained their ability to interact with dTLK (data not shown), loss of all three Serines completely abolished dTLK-mediated phosphorylation (Figure 1C), indicating that S211 and S213 serve as minor additional phosphorylation sites.

Next, we performed a similar mapping of phosphorylation sites on human hASF1a and hASF1b with baculovirus expressed hTLK2 (Figure 1A). We found that only S192A in hASF1a and S198A in hASF1b significantly affected phosphorylation by hTLK2 (Figure 1E, F). Alignment of the major phosphorylation sites yielded a (D/E)-N-S-(L/M) consensus motif for the TLK target site (Figure 1D). As mentioned above, the dASF1 minor phosphorylation sites S211 and S213 are located in a similar motif (Figure 1D).

### TLK-Mediated Control of ASF1 Cellular Levels

**Figure 2:**
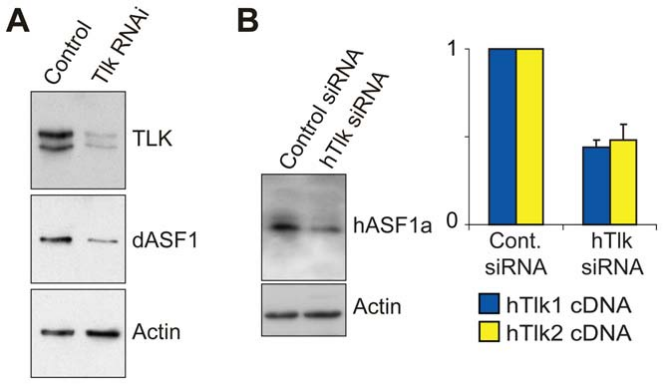
TLK activity is required for ASF1 stability *in vivo*. (**A**) Depletion of dTLK from Drosophila S2 cells leads to decreased dASF1 levels. S2 cells were either mock treated or incubated with dsRNA directed against dTLK. Whole-cell extracts were prepared and analyzed by Western blotting with indicated antibodies. Actin serves as a loading control. (**B**) siRNA for hTLK1 and hTLK2 affects hASF1a stability in HeLa cells. Whole-cell extracts from control or siRNA treated cells were analyzed by Western blotting with anti-hASF1a and anti-Actin antibodies. Efficiency of siRNA of hTLK1 (blue) and hTLK2 (yellow) was confirmed by RT-qPCR normalized to control siRNA (right panel).

To examine the role of ASF1 phosphorylation *in vivo*, we performed RNAi-mediated depletion of dTLK in Drosophila S2 cells and siRNA-mediated depletion of both hTLK1 and hTLK2 genes in HeLa cells. Reduced TLK levels resulted in significant reductions in dASF1 and hASF1a protein levels (Figure 2A, B). These results suggested that phosphorylation by TLK might stabilize ASF1.

**Figure 3:**
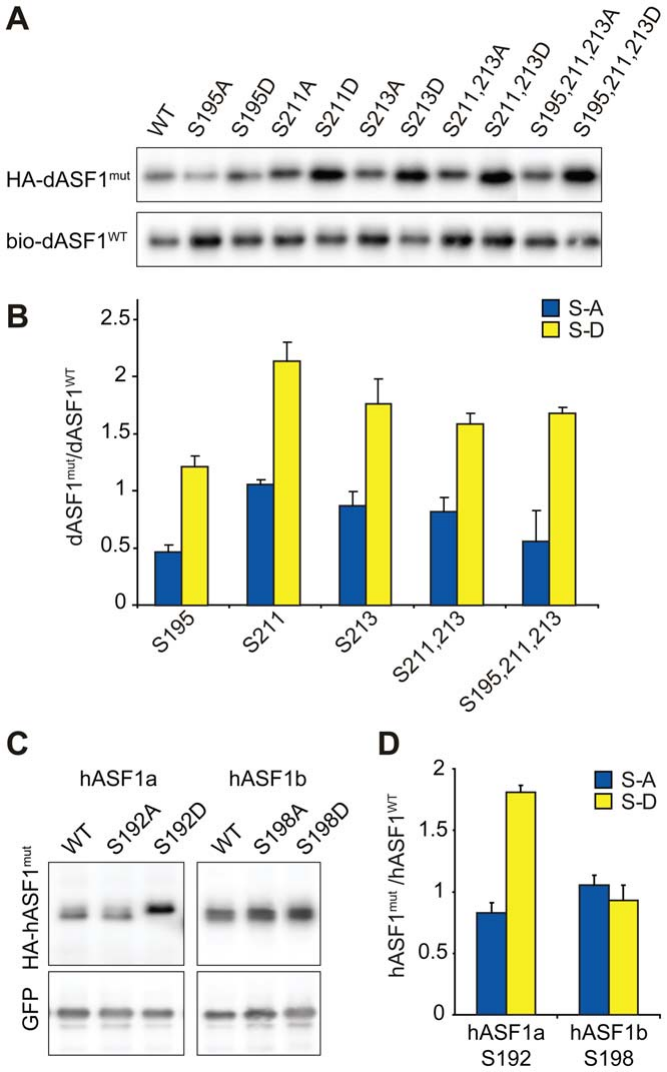
Mutations in TLK phosphorylation sites affect ASF1 protein levels. (**A**) Mutated dASF1 proteins fused to HA-tag were co-expressed with wild-type bio-tagged dASF1 in S2 cells and revealed by immunoblotting. Mutated Serines within dASF1 protein are indicated. (**B**) Levels of mutant HA-dASF1 proteins were quantified and normalized to bio-dASF1wt protein level and compared to the same ratio obtained for HA-dASF1wt. The graph shows the mean for three experiments and error bars show standard errors of the mean (SEM). Mutated Serines are indicated (S-A mutations – blue bars, S-D – yellow bars). (**C, D**) Mutated HA-hASF1 proteins were co-expressed with GFP in HEK293T cells. Representative immunoblots are shown (**C**) and HA-hASF1 protein levels were analyzed as above (**D**) using GFP levels as a reference. The graph shows the mean for three experiments and error bars show SEM. Mutated Serines are indicated (S-A mutations – blue bars, S-D – yellow bars).

To investigate this further, we tested the effect of introducing substitution mutants that no longer could be phosphorylated. For this, we expressed both wild-type and mutant dASF1 proteins in S2 cells (Figure 3A) and compared their steady-state levels. A significant decrease in protein level was observed for mutants S195A and S195A/S211A/S213A (Figure 3A, B). Conversely, phosphorylation-mimicking mutations S211D, S213D, S211D/S213D and S195D/S211D/S213D resulted in significant protein accumulation (Figure 3A, B). Similarly, we tested the effect of mutations in hASF1a and hASF1b on protein stability. While the hASF1a S192A mutation had a modest effect on hASF1a stability, the phosphorylation mimicking S192D mutation showed significantly higher protein levels (Figure 3C, D). Neither of the hASF1b mutations affected protein levels, suggesting a lack of posttranslational control by TLKs (Figure 3C, D). However, hASF1b levels have recently been shown to be controlled by the cell-cycle regulated E2F transcription factors [20]. Together, these results suggest that phosphorylation of dASF1 and hASF1a by TLKs stabilizes the ASF1 proteins.

### ASF1 Phosphorylated by TLK Escapes from Proteolysis

**Figure 4:**
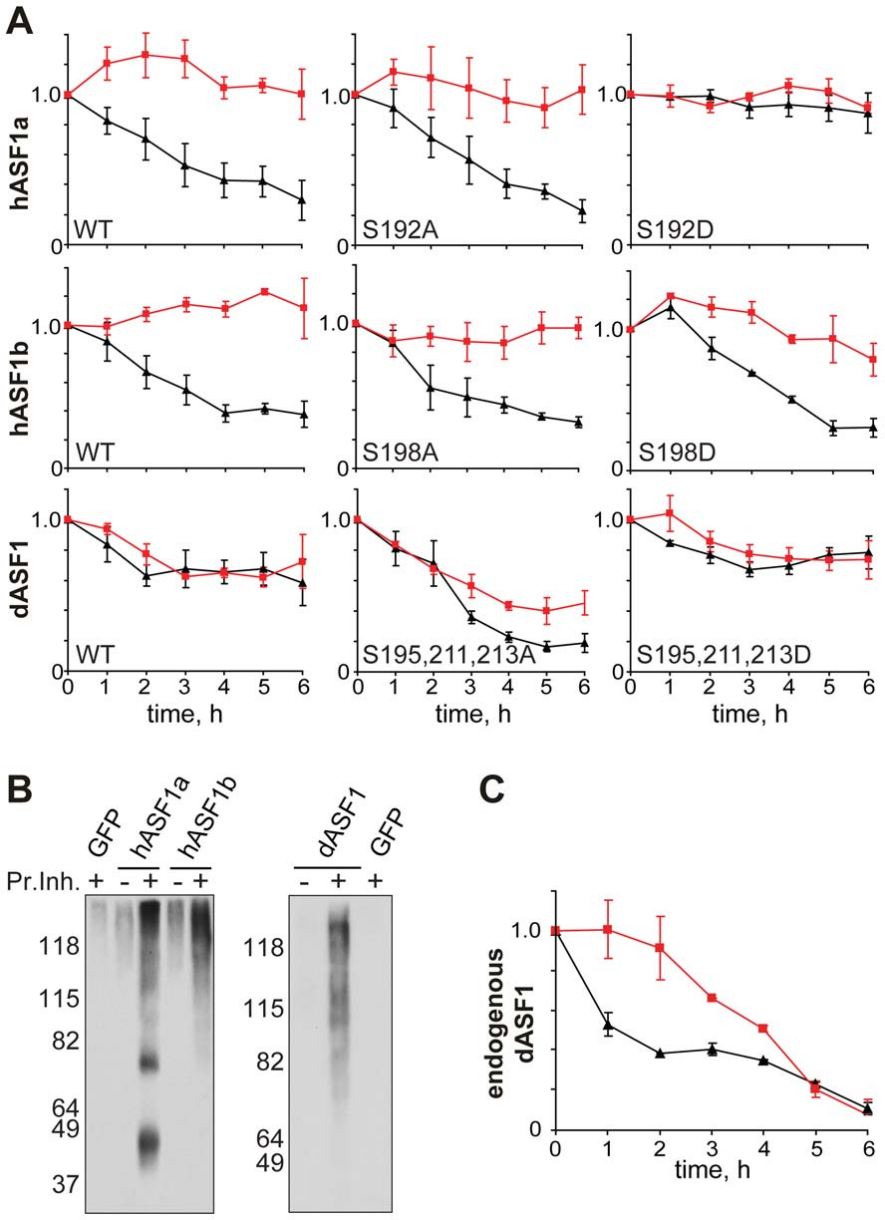
Phosphorylation of ASF1 hampers proteasome-dependent degradation. (**A**) hASF1 and dASF1 wild type or mutant proteins were expressed in HEK293T and S2 cells respectively. Protein synthesis was blocked by cycloheximide in cells incubated with (red curves) or without (black curves) proteasome inhibitors lactacistin and MG132. ASF1 protein levels were quantified as in Figure 3 by immunoblotting for hASF1 (upper panels) and dASF1 (lower panel) every hour from 0 to 6 h and normalized to actin. All experiments were repeated at least three times and error bars show SEM. (**B**) HA-hASF1 and HA-dASF1 proteins or GFP were expressed in HEK293T cells. Cells were either treated (+Pr.Inh.) or not (-Pr.Inh.) with proteasome inhibitors lactacistine and MG132. Whole cell extracts were incubated with anti-HA beads and pulled-down proteins were analyzed on a western blot with anti-ubiquitin antibody. High-molecular weight smear represents poly-ubiquitinated ASF1. (**C**) Degradation of endogenous dASF1 dependents only partially on proteasome pathway. Protein synthesis was blocked by cycloheximide in S2 cells incubated with (red curves) or without (black curves) proteasome inhibitors lactacistin and MG132. Samples were collected every hour from 0 to 6 h. dASF1 protein was immunoblotted and quantified as above. Data are represented as mean of three experiments +/− SEM.

Next, we decided to examine the degradation rates of wild-type and mutant ASF1 proteins. After a cycloheximide block, the levels of hASF1 wild-type proteins transfected into HEK293T cells drop more then two-fold within 3 hours (Figure 4A). In contrast, the level of the hASF1a S192D mutant protein remains almost unchanged for six hours after a cycloheximide block (Figure 4A). Likewise, dASF1 S195A/S211A/S213A mutant protein expressed in Drosophila S2 cells degrades faster when compared to the wild type dASF1 or S195D/S211D/S213D mutant protein (Figure 4A).

To investigate the mechanism of ASF1 degradation, we performed the same experiments in the presence of the proteasome inhibitors lactacystin and MG132. For the hASF1s, incubation with proteasome inhibitors leads to a significant stabilization of hASF1a and hASF1b and a concomitant accumulation of polyubiquitinated ASF1 (Figure 4A, B). Although dASF1 can also be polyubiquitinated (Figure 4B), we observed only modest decrease in degradation rates of the endogenous dASF1 (Figure 4C) and dASF1 S195A/S211A/S213A mutant protein upon incubation with proteasome inhibitors (Figure 4A). This suggests that other protein degradation pathways could be involved in the control of dASF1 stability, in addition to proteasome-mediated proteolysis.

## Discussion

In conclusion, our results uncover a role for the cell-cycle regulated Tousled-like kinases in post-translational regulation of ASF1. We show that the major TLK phosphorylation sites are located in non-conserved C-terminal part of Drosophila and human ASF1 proteins within (D/E)-N-S-(L/M) consensus motif. Phosphorylation of ASF1 by TLKs regulates positively dASF1 and hASF1a protein levels and hampers proteasome-dependent degradation of hASF1a. In Drosophila, dASF1 degradation is only partially controlled by proteasome-dependent proteolysis and other degradation pathways could be involved, but nonetheless phosphorylation by dTLK results in significant stabilization of dASF1. Finally, hASF1b stability does not appear to depend on phosphorylation by TLKs, but recently it has been shown that hASF1b transcription is controlled by the cell-cycle regulated E2F transcription factors [20]. Together, these results suggest that ASF1 cellular levels are under tight control by either transcriptional or post-translational mechanisms.

ASF1 and TLK activities are critical for chromatin replication and progression through S phase [5], [7], [17], [18], [21]. Both TLK-dependent phosphorylation and E2F-mediated expression coincide with S phase [22], [23]. Therefore, it is interesting to speculate that similarly to yeast [24], regulation of Drosophila and human ASF1 cellular levels may be linked to cell cycle progression, albeit by different mechanisms.

Major TLK phosphorylation sites are located within non-conserved C-terminal part of Drosophila and human ASF1 proteins. However, it has been shown that in yeast constitutive expression of conserved N-terminal part is sufficient to recapitulate most of the ASF1 functions *in vivo* [25], [26], [27]. Therefore, we believe that the major function for TLK-mediated phosphorylation of ASF1 is in fine-tuning of ASF1 cellular levels, rather than in modifying of its activity.

## Materials and Methods

### Proteins Purification, In Vitro Phosphorylation and Mass Spectrometry Analysis

The constructs for recombinant protein purification from bacteria or baculovirus system were based on pGEX-4T and pFastBacHT plasmid series (Invitrogen) respectively. The constructs used for protein expression in Drosophila and human cells were based on pRMH3-BLPR containing BirA signal [28], pMT/V5-His-B (Invitrogen), or pcDNA3.1(+) vector (Invitrogen). Point mutations were made with QuikChangeII kit (Stratagene). Further details can be obtained upon request.

GST-ASF1 protein fusions were expressed and purified as described [15]. HT-TLKs were purified using Bac-to-Bac baculovirus expression system (Invitrogen), and phosphorylation of ASF1 by TLKs was performed as described [17]. For mass spectrometric analysis phosphopeptides were selectively enriched for on TiO_2_ column [29] and analyzed on an LTQ-Orbitrap (Thermo).

### Antibodies and Immunological Procedures

Immunoblotting were performed using standard procedures with the following antibodies: anti-dASF1, anti-dTLK [17], anti-hASF1a [18], anti-beta-Actin (Abcam), anti-GFP (Roche), anti-HA.11 (Covance) and anti-Ubiquitin P4D1 (Santa Cruz). Biotinylated proteins were detected with VECTASTAIN ABC kit (Vector laboratories). Proteins were visualized with Lumi-Light^PLUS^ chemiluminescent substrate (Roche) and detected with Syngene GeneGnome CCD camera setup.

### Cell Culture, RNAi, siRNA and Analysis of ASF1 Protein Levels

S2 cells were cultured in Schneider's medium (Invitrogen 21720–024) supplied with 10% FBS and Penicillin/Streptomycin mixture at 25°C [30]. HEK293T and HeLa cells were cultured in DMEM (Invitrogen 10938–025) supplied with 10% FBS and penicillin/streptomycin mixture at 37°C in CO_2_ incubator as described [30]. RNAi in S2 cells was performed as described [31]. Dharmacon siGENOME SMARTpool reagents M-004174-00-0005 and M-005389-00-0005 - 100µM of each or 200µM of the control pool D-001206-13-05 were used for siRNA hTLK1,2 in HeLa cells for 72 hours.

FuGene6 transfectin reagent (Promega) was used for the transient transfection of cell cultures. Expressed proteins were assayed 48 h post-induction for metallothioneine promoter constructs or 48 h post-transfection for other constructs by chemiluminescent western-blotting and quantified using Syngene GeneSnap software. To inhibit proteasome-dependent degradation cells were incubated for 16 h in the presence of 5µM Lactacystine and 10 mM MG-132 (Sigma), and protein synthesis was blocked using 50µM of cycloheximide [30].

## References

[1] De Koning L, Corpet A, Haber JE, Almouzni G (2007). Histone chaperones: an escort network regulating histone traffic.. Nat Struct Mol Biol.

[2] Eitoku M, Sato L, Senda T, Horikoshi M (2008). Histone chaperones: 30 years from isolation to elucidation of the mechanisms of nucleosome assembly and disassembly.. Cell Mol Life Sci.

[3] Park YJ, Luger K (2008). Histone chaperones in nucleosome eviction and histone exchange.. Curr Opin Struct Biol.

[4] Moshkin YM, Kan TW, Goodfellow H, Bezstarosti K, Maeda RK (2009). Histone chaperones ASF1 and NAP1 differentially modulate removal of active histone marks by LID-RPD3 complexes during NOTCH silencing.. Mol Cell.

[5] Tyler JK, Adams CR, Chen SR, Kobayashi R, Kamakaka RT (1999). The RCAF complex mediates chromatin assembly during DNA replication and repair.. Nature.

[6] Tyler JK, Collins KA, Prasad-Sinha J, Amiott E, Bulger M (2001). Interaction between the Drosophila CAF-1 and ASF1 chromatin assembly factors.. Mol Cell Biol.

[7] Groth A, Corpet A, Cook AJ, Roche D, Bartek J (2007). Regulation of replication fork progression through histone supply and demand.. Science.

[8] Zhang R, Chen W, Adams PD (2007). Molecular dissection of formation of senescence-associated heterochromatin foci.. Mol Cell Biol.

[9] Green EM, Antczak AJ, Bailey AO, Franco AA, Wu KJ (2005). Replication-independent histone deposition by the HIR complex and Asf1.. Curr Biol.

[10] Das C, Lucia MS, Hansen KC, Tyler JK (2009). CBP/p300-mediated acetylation of histone H3 on lysine 56.. Nature.

[11] Chen CC, Carson JJ, Feser J, Tamburini B, Zabaronick S (2008). Acetylated lysine 56 on histone H3 drives chromatin assembly after repair and signals for the completion of repair.. Cell.

[12] Takahata S, Yu Y, Stillman DJ (2009). FACT and Asf1 regulate nucleosome dynamics and coactivator binding at the HO promoter.. Mol Cell.

[13] Gkikopoulos T, Havas KM, Dewar H, Owen-Hughes T (2009). SWI/SNF and Asf1p cooperate to displace histones during induction of the saccharomyces cerevisiae HO promoter.. Mol Cell Biol.

[14] Adkins MW, Williams SK, Linger J, Tyler JK (2007). Chromatin disassembly from the PHO5 promoter is essential for the recruitment of the general transcription machinery and coactivators.. Mol Cell Biol.

[15] Moshkin YM, Armstrong JA, Maeda RK, Tamkun JW, Verrijzer P (2002). Histone chaperone ASF1 cooperates with the Brahma chromatin-remodelling machinery.. Genes Dev.

[16] Goodfellow H, Krejci A, Moshkin Y, Verrijzer CP, Karch F (2007). Gene-specific targeting of the histone chaperone asf1 to mediate silencing.. Dev Cell.

[17] Carrera P, Moshkin YM, Gronke S, Sillje HH, Nigg EA (2003). Tousled-like kinase functions with the chromatin assembly pathway regulating nuclear divisions.. Genes Dev.

[18] Sillje HH, Nigg EA (2001). Identification of human Asf1 chromatin assembly factors as substrates of Tousled-like kinases.. Curr Biol.

[19] Mello JA, Sillje HH, Roche DM, Kirschner DB, Nigg EA (2002). Human Asf1 and CAF-1 interact and synergize in a repair-coupled nucleosome assembly pathway.. EMBO Rep.

[20] Hayashi R, Goto Y, Tanaka R, Oonogi K, Hisasue M (2007). Transcriptional regulation of human chromatin assembly factor ASF1.. DNA Cell Biol.

[21] Groth A, Ray-Gallet D, Quivy JP, Lukas J, Bartek J (2005). Human Asf1 regulates the flow of S phase histones during replicational stress.. Mol Cell.

[22] Sillje HH, Takahashi K, Tanaka K, Van Houwe G, Nigg EA (1999). Mammalian homologues of the plant Tousled gene code for cell-cycle-regulated kinases with maximal activities linked to ongoing DNA replication.. Embo J.

[23] Berckmans B, De Veylder L (2009). Transcriptional control of the cell cycle.. Curr Opin Plant Biol.

[24] Le S, Davis C, Konopka JB, Sternglanz R (1997). Two new S-phase-specific genes from Saccharomyces cerevisiae.. Yeast.

[25] Daganzo SM, Erzberger JP, Lam WM, Skordalakes E, Zhang R (2003). Structure and function of the conserved core of histone deposition protein Asf1.. Curr Biol.

[26] Sanematsu F, Takami Y, Barman HK, Fukagawa T, Ono T (2006). Asf1 is required for viability and chromatin assembly during DNA replication in vertebrate cells.. J Biol Chem.

[27] Umehara T, Chimura T, Ichikawa N, Horikoshi M (2002). Polyanionic stretch-deleted histone chaperone cia1/Asf1p is functional both in vivo and in vitro.. Genes Cells.

[28] Mito Y, Henikoff JG, Henikoff S (2005). Genome-scale profiling of histone H3.3 replacement patterns.. Nat Genet.

[29] Pinkse MW, Uitto PM, Hilhorst MJ, Ooms B, Heck AJ (2004). Selective isolation at the femtomole level of phosphopeptides from proteolytic digests using 2D-NanoLC-ESI-MS/MS and titanium oxide precolumns.. Anal Chem.

[30] Bajpe PK, van der Knaap JA, Demmers JA, Bezstarosti K, Bassett A (2008). Deubiquitylating enzyme UBP64 controls cell fate through stabilization of the transcriptional repressor tramtrack.. Mol Cell Biol.

[31] Worby CA, Simonson-Leff N, Dixon JE (2001). RNA interference of gene expression (RNAi) in cultured Drosophila cells.. Sci STKE 2001: PL1.

